# Antegrade common femoral artery access site closure using the MANTA vascular closure device

**DOI:** 10.1016/j.radcr.2020.08.053

**Published:** 2020-09-07

**Authors:** Wouter Stomp, Daniël Eefting, Jan van Schaik, Davy R. Sudiono, Rutger W. van der Meer

**Affiliations:** a Department of Radiology, Leiden University Medical Center, P.O. Box 9600, 2300 RC, Leiden, the Netherlands; b Department of Surgery, Leiden University Medical Center, 2300 RC, Leiden, the Netherlands; c Department of Surgery, Haaglanden Medical Center, 2501 CK, The Hague, the Netherlands; d Department of Radiology, Haaglanden Medical Center, 2501 CK, The Hague, the Netherlands

**Keywords:** Interventional radiology, Antegrade access, Vascular closure device, Common femoral artery

## Abstract

In antegrade peripheral endovascular procedures, the use of covered stents may require a large sheath size, which precludes the use of regular closure devices. The MANTA vascular closure device is a collagen plug-based vascular closure device for large bore percutaneous arterial interventions, which is normally used to close retrograde vascular access sites. We describe successful antegrade common femoral access site closure with the MANTA vascular closure device in 2 patients, a 68-year-old male and an 89-year-old male, both with a popliteal artery aneurysm which was treated by percutaneous endovascular stentgraft placement. Use of the MANTA vascular closure device simplifies large-bore antegrade common femoral artery access and avoids the need for surgical artery cutdown.

## Introduction

Large-bore vascular closure devices are increasingly used to close the common femoral artery access site in retrograde endovascular procedures such as EVAR and TAVI. The MANTA vascular closure device (Teleflex, PA, USA) is a collagen plug-based vascular closure device for large bore percutaneous arterial interventions. It can be used to close 10–20F vascular access sites (12–25F outer diameter) [1]. Although antegrade peripheral arterial procedures are usually performed through smaller sized vascular access sheaths, the use of covered stents may require a larger sheath size, which precludes the use of regular closure devices. We describe 2 cases in which an antegrade femoral artery access site was successfully closed with the MANTA device. This report was exempted from review by the local medical research ethics committee.

The first patient is a 68-year-old male without any relevant past medical history, who presented in the emergency department with acute right lower leg pain. At physical examination the lower leg was cold and pale. Arterial doppler signals were present in the popliteal artery, but not in the crural arteries. Motor and sensory functions were normal. CT angiography demonstrated a fusiform popliteal artery aneurysm with a maximum diameter of 30 mm extending over a length of 70 mm, with extensive mural thrombus. Multiple embolic thrombi in the anterior tibial artery, peroneal artery and posterior tibial artery were noted, with no contrast enhancement distally. Intra-arterial thrombolysis was initiated, and after 2 days the emboli had partially resolved. The plantar arteries were sufficiently perfused through collaterals so thrombolysis was discontinued.

**Fig. 1:**
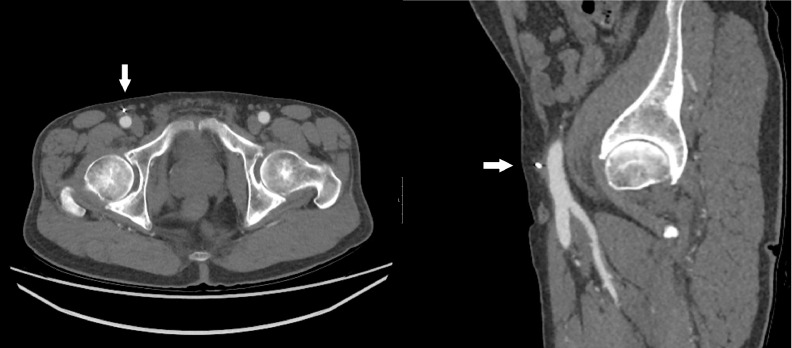
Follow-up CT angiography of the first patient 3 months after the procedure. Axial and sagittal reconstructions show a patent right common femoral artery with minimal wall irregularity following resorption of the endovascular anchor. Outside the artery the collagen plug and stainless steel suture lock are visible (arrows).

This patient preferred the popliteal aneurysm to be treated endovascularly. Antegrade access to the right common femoral artery was obtained using a standard Seldinger technique under ultrasound guidance. As recommended by the manufacturer, preprocedural measurement of the artery depth from the skin was performed using the provided 8F puncture location dilator. Subsequently the dilator was exchanged for a 12F sheath inserted in an antegrade direction. Two overlapping Viabahn stentgrafts (9 × 100 mm and 11 × 100 mm, GORE, DE) were placed to exclude the popliteal aneurysm. At the end of the procedure, a 14F MANTA device was deployed according to the same procedure as recommended by the manufacturer for retrograde access site closure. In short, the procedural sheath is exchanged for the dedicated MANTA sheath and the closure device is inserted. After withdrawing the assembly according to the predetermined artery depth, a resorbable anchor is released within the vascular lumen and a collagen plug is deployed outside the artery, operated by a simple toggle system. The anchor and the collagen plug are secured by a polymer suture and stainless steel suture lock, forming a "sandwich" across the arterial entry site. Arterial hemostasis was achieved instantaneously.  The post-procedural period was complicated by acute stentgraft thrombosis, which resolved after intra-arterial thrombolysis. No access-site related complications occurred, and a follow-up CT angiography 3 months later showed a patent access vessel with minimal wall irregularity (Fig. 1).

The second patient was an 89-year-old male with an extensive vascular history including EVAR for an infrarenal abdominal aortic aneurysm, and a left popliteal artery aneurysm which was surgically reconstructed. He had a known right popliteal artery aneurysm with a diameter of 67 mm for which he previously declined treatment. Subsequently he presented with pain and a large hematoma in the right popliteal fossa. CT angiography showed enlargement of the popliteal aneurysm with an irregular border on the medial side with adjacent fat infiltration, which was interpreted as a symptomatic aneurysm with rupture or impending rupture of the aneurysm. Antegrade access to the common femoral artery was obtained under ultrasound guidance. Via a 12F sheath 2 overlapping Viabahn stentgrafts (9 × 100 mm and 10 × 150 mm) were placed to exclude the popliteal aneurysm. Angiography showed minimal residual filling of the aneurysm sac through collateral vessels. A 14F MANTA device was used to close the access site. No postprocedural complications occurred.

## Discussion

Several studies have established the safety and efficacy of small-bore vascular closure devices after antegrade common femoral artery access, with a moderately higher rate of device failure with conversion to manual compression than in retrograde access [2]. We describe the successful closure of a large bore antegrade common femoral artery access with the MANTA vascular closure device in 2 patients. Without the use of a closure device, these procedures would have required a surgical cutdown for arterial access. Closure of antegrade access sites is outside the manufacturer's instructions for use; none of the large-bore vascular closure devices currently available on the market are approved for this purpose. One other report was found on the antegrade use of a large-bore vascular closure device, in which a 10F Prostar XL (Abbott, IL) suture-mediated closure device was placed after antegrade common femoral artery access with a 11F sheath [3]. In addition, the 6F ProGlide (Abbott, IL) suture-mediated closure device has been used to close the antegrade access after the use of arterial sheaths with a size of up to 12F [4]. Compared to suture-mediated closure devices, the MANTA device does not need pre-closure and there is no need for the deployment of multiple devices with larger sheath sizes. Following placement of the MANTA device, it takes 6 months for the intra-arterial bioresorbable polymer toggle and extra-vascular bovine collagen pad to resorb, after which only a polymer suture and stainless steel suture lock remain at the access site. In conclusion, percutaneous antegrade common femoral access site closure with the MANTA vascular closure device simplifies antegrade large-bore common femoral artery access and diminishes the potential complications caused by surgical artery cutdown.

## Compliance with Ethical Standards

### Funding

This research did not receive any specific grant from funding agencies in the public, commercial, or not-for-profit sectors.

### Ethical approval

All procedures performed in studies involving human participants were in accordance with the ethical standards of the institutional and/or national research committee and with the 1964 Helsinki declaration and its later amendments or comparable ethical standards.

### Informed consent

This study has obtained IRB approval from METC Leiden Den Haag Delft and the need for informed consent was waived.

### Consent for publication

Consent for publication was obtained for every individual person's data included in the study.

All patients described in this case report provided written, informed consent for publication of their case.

## Conflict of interest

None.
